# Predicting and comparing postoperative infections in different stratification following PCNL based on nomograms

**DOI:** 10.1038/s41598-020-68430-3

**Published:** 2020-07-09

**Authors:** Enyan Jiang, Haixiang Guo, Bowei Yang, Pei Li, Prashant Mishra, Tongxin Yang, Yuhang Li, Haifeng Wang, Yongming Jiang

**Affiliations:** Yunnan Key Laboratory of Urology, Yunnan Urology Speciality Hospital, The Second Affiliated Hospital of Kunming Medical University, Kunming, Yunnan People’s Republic of China

**Keywords:** Immunology, Microbiology, Physiology

## Abstract

To discuss the mechanisms of infection complications in different degrees after percutaneous nephrolithotomy (PCNL) through predicting and comparing post-PCNL infections based on nomograms, a retrospective cohort study was conducted among 969 cases who underwent PCNL from Dec 5, 2016 to Dec 25, 2017 in Kunming, Yunnan Province. We examined clinical features, urine routine, blood routine, blood biochemistry, imaging studies and operative information and recorded the examination results before surgery for univariate and multivariate logistic regression. We applied receiver operating characteristic curves, calibration curves, accuracy, specificity, sensitivity, positive predictive value and negative predictive value to evaluate and compare the models. Nomograms were used to visualize the different degrees of postoperative infection complications. The risk scores of the three groups were compared by diabetes mellitus distribution. Our results suggest that the more severe the infection is, the more accurate the model predicts and that the occurrence of severe infection mostly is related to the patients' homeostasis. Hence, we developed an online post-PCNL sepsis dynamic nomogram which can achieve visualization and dynamically predict the incidence of sepsis in postoperative patients.

## Introduction

Kidney stones, one of the most common urologic diseases, show an upward trend annually^1^, especially in Southwest China where the incidence rate is higher than those in other parts of the country^2^. PCNL is used as the standard method in the treatment of upper urinary tract stone > 2 cm^3^ with minimal invasion and a faster and higher stone clearance rate^4^. However, complications are reported including fever (21.0–32.1%), blood transfusion (11.2–17.5%), extravasation (7.2%) and septicemia (0.3–4.7%)^5^. Infection, as a major complication, is graded into fever, systemic inflammatory response syndrome (SIRS), and sepsis according to the severity. Previous studies have reported the risk factors on the severity of infectious complications, such as gender, nephrostomy^6^, preoperative positive urine culture^7^, stone size^8^, age, diabetes mellitus and complex stones^9^. Risk factors of SIRS include PCNL operation history, stone size, degree of hydronephrosis, complex stones, preoperative positive urine culture, perfusion pressure, and neurogenic bladder and the use of antibiotics^10–13^. Risk factors of sepsis include stone burden, infectious stone, the number of tracts, preoperative positive urine culture, leukopenia, creatinine and operation time^14–17^. Nonetheless, most studies only discussed one or two infection outcomes and did not compare similarities and differences of infection concurrency between different degrees in the same samples. In this study, we established models to predict and to study the infection complications of different degrees after PCNL, and compared the differences between the models to illustrate the different infection mechanisms and the clinical significance. Finally, we developed online application to achieve visualization and dynamically predict the incidence of postoperative infection.

## Methods

### Patient selection

From Dec. 5, 2016 to Dec. 25, 2017, a total number of 1,003 patients with renal stones underwent PCNL in the Second Affiliated Hospital of Kunming Medical University, Yunnan Province. Nineteen (1.9%) patients were excluded because of preoperative fever and 15 (1.5%) were excluded because of incomplete data. Finally, 969 patients were included in this study. We conducted a retrospective study and obtained patients’ demographic data as preoperative information. These information included: (1) Demographic data: gender, age, body mass index (BMI), hypertension and DM; (2) Urine routine: urine red blood cell (UR), urine white blood cell (UW), urine bacteria (UB), urine nitrite (UNIT) (0: < 0.08 mg/dL, 1: 0.08–0.2 mg/dL, 2: > 0.2 mg/dL) and urine pH (UpH); (3) Urine culture (UC); (4) Blood routine: serum white blood cell (WBC), serum neutrophils (N), serum lymphocytes (L), platelet (PLT), and hemoglobin (HGB); (5) Blood biochemistry: albumin (ALB), creatinine (Cr), uric acid (UA), fasting blood sugar (GLU), serum potassium (K), serum calcium (Ca), serum phosphorus (P), and magnesium (Mg); (6) Imaging study: size (maximum diameter of stone), position in KUB (kidney/ureter/bladder) and left or right, computed tomography hounsfield unit (CT-HU, the mean CT value of the largest stone), multiple stones and hydronephrosis; (7) Operative information: operation time and sheath size. Since more than 50% of the data of serum procalcitonin (PCT), C-reactive protein (CRP) and interleukin-6 (IL-6) were missing, they were excluded considering the accuracy of the study. The chest radiographs were not included as the reports did not show any symptoms of infection. Preoperative variables, such as antibiotic usage, urinary anatomical abnormalities, stent size, etc., were also not included.

Preoperative patients used antibiotics routinely at least 1 day. The patients with (1) UW ≥ 10 per high-power field in the re-suspended sediment of a centrifuged aliquot of urine or (2) UC positive received antibiotics for 3–14 days (mean preoperative preparation 6.60 ± 3.21 days). If the review results turned negative, surgery was performed.

The sheath used to establish PCNL tract was Fr 18–24. Holmium laser was used for lithotripsy with a maximum energy of 60 W, after lithotripsy stent and nephrostomy tube were placed. Blood tests and vital signs were performed immediately after surgery (repeated tests for severe patients). Urine routine was conducted in the next morning.

1. Postoperative fever was defined as the body temperature over 38 °C during hospital stay after PCNL. 2. Postoperative SIRS criteria referred to American College of Chest Physicians (ACCP) and the Society of Critical Care Medicine (SCCM) in 2001 (At least two conditions were met after PCNL): (1) hyperthermia (> 38.0 °C) or thermia (< 36 °C); (2) heart rate > 90 bpm; (3) respiratory rate > 20 breaths/min or PaCO2 < 32 mmHg; (4) white blood cell count > 12,000 cells/μL or < 4,000 cells/μL^18^. 3. Sepsis was defined as two or more criteria of the quick sepsis-related organ failure assessment (qSOFA) were met: respiratory rate of ≥ 22 breaths/min, altered consciousness (Glasgow Coma Scale score of < 13), and systolic blood pressure of ≤ 100 mmHg^19^. The postoperative interval was 1.47 ± 1.40 days, 1.94 ± 1.26 days and 4.00 ± 4.08 days for fever, SIRS and sepsis in our study, respectively.

The study protocol conforms to the ethical guidelines of the 1975 Declaration of Helsinki as reflected in a prior approval by the Ethics Committee of the Second Affiliated Hospital of Kunming Medical University. Moreover, we confirmed that all experiments were performed in accordance with relevant guidelines and regulations. All participants gave their informed consent.

### Statistical analysis

Shapiro–Wilk test detected that all variables were not normally distributed and showed in the form of median [IQR]. The categorical variables were expressed in percentage (%). Mann–Whitney U test compared non-normal data. The chi-square test, or Fisher's exact probability method, was used to compare differences between categorical variables. The variables in the univariate logistic regression whose significance was less than 0.05 could be included in the multivariate logistic regression (Supplement Tables S2, S4 and S6). The selection of the final prediction model was performed with a backward step down selection process with Akaike information criterion (AIC). Generalized collinearity diagnostics were carried out in the current study to evaluate multicollinearity between variables (Supplement Tables S3, S5 and S7). The significant variables in multivariate regression were included in the nomogram. The prediction of the models was evaluated by concordance index (C-index) which was equivalent to area under curve (AUC) in logistic regression compared by Delong test in ROC. Calibration curves were used to visualize the consistency between the predicted results and the observed results. The prediction results were evaluated with accuracy, specificity, sensitivity, PPV and NPV. The software used in data analysis was R version 3.6.3 (https://www.r-project.org/). A two-tailed test was performed and a *p* value less than 0.05 was considered significant.

## Results

From Dec 5, 2016 to Dec 25, 2017, a total number of 1,003 patients underwent PCNL. Thirty-four excluded cases did not meet selected standards. The demographic data and clinical characteristics of 969 cases were displayed in Supplement Table S1. According to the severity of the outcomes, the patients were divided into three groups: 219 (22.6%) cases with fever, 166 (17.1%) cases with SIRS and 25 (2.6%) cases with sepsis.

As shown in Table 1, the odds ratios (ORs) and significance of the variables were displayed in multivariate logistic regression. In fever model, the variables with high significance were UW, UNIT and those with no significance were UC, HGB, P and Staghorn. In SIRS model, the variables with high significance were UC, UNIT and Operation time, and the ORs were no significantly for UW, PLT and Staghorn. In sepsis model, UC, UNIT, UpH, Ca and Operation time were significant. Gender and Staghorn were not significant.

**Table 1:** Multivariate logistic regression of post-PCNL fever, SIRS and sepsis.

Models	Characteristic	Adj. OR (95% CI)	*P* (Wald's test)	*P* (LR-test)
Post-PCNL fever	UW (cont. var.)	1.087 (1.021, 1.156)	0.009*	0.006*
	UNIT: ref. = 0			< 0.001*
	1	0.987 (0.358, 2.718)	0.979	
	2	2.546 (1.582, 4.097)	< 0.001*	
	UC: Positive versus negative	1.480 (0.994, 2.204)	0.054	0.057
	HGB (cont. var.)	0.9915 (0.9828, 1.0002)	0.055	0.055
	*P* (cont. var.)	2.045 (0.934, 4.478)	0.074	0.075
	Staghorn: Yes versus no	1.457 (0.998, 2.127)	0.052	0.055
Post-PCNL SIRS	UW (cont. var.)	1.056 (0.995, 1.121)	0.073	0.064
	UC: Positive versus negative	1.887 (1.231, 2.893)	0.004*	0.004*
	UNIT: ref. = 0			< 0.001*
	1	1.100 (0.373, 3.241)	0.863	
	2	2.880 (1.76, 4.712)	< 0.001*	
	PLT (cont. var.)	1.002 (0.9997, 1.0044)	0.085	0.088
	Staghorn: Yes versus no	1.484 (0.981, 2.246)	0.062	0.066
	Operation time (cont. var.)	1.0046 (1.0011, 1.0082)	0.011*	0.012*
Post-PCNL sepsis	Gender: Male versus female	0.446 (0.173, 1.149)	0.094	0.087
	UNIT: ref. = 0			0.046*
	1	0.816 (0.089, 7.488)	0.058	
	2	2.794 (1.060, 7.362)	0.038*	
	UC: Positive versus negative	4.985 (1.713, 14.511)	0.003*	0.002*
	Staghorn: Yes versus no	2.417 (0.970, 6.021)	0.058	0.065
	UpH (cont. var.)	1.985 (1.016, 3.879)	0.045*	0.046*
	Ca (cont. var.)	0.004 (0, 0.146)	0.003*	0.003*
	Operation time (cont. var.)	1.0092 (1.0009, 1.0176)	0.030*	0.034*

*Values indicate statistical significance (*P* < 0.05).

UW, urine white blood cell; UNIT, urine nitrite; UC, urine culture; HGB, hemoglobin; P, serum phosphorus; PLT, platelet; UpH, urine pH; Ca, serum calcium; SIRS, systemic inflammatory response syndrome; cont. var., continue variable; ref., reference.

**Fig.1:**
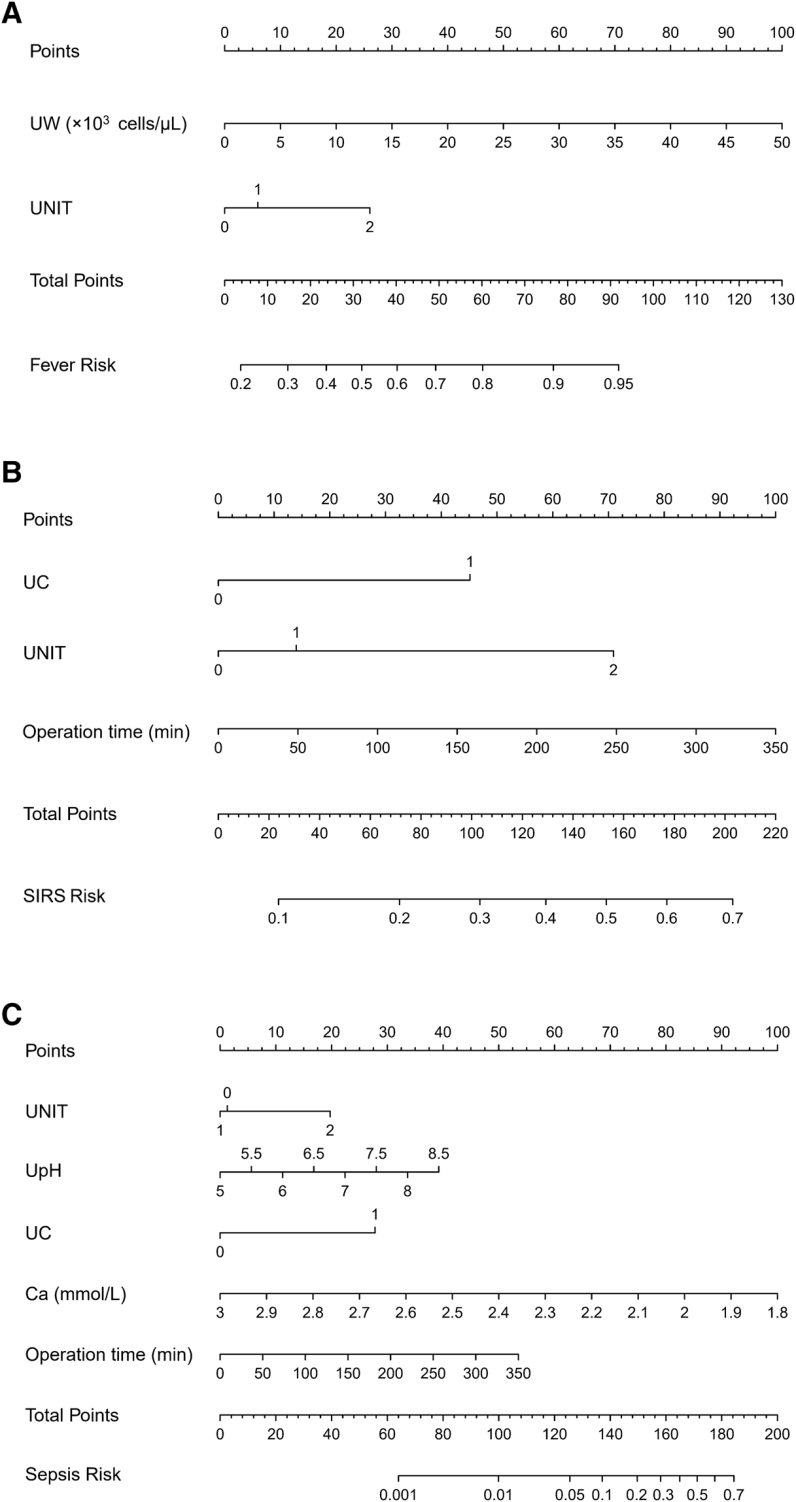
Nomograms developed for post-PCNL (**A**) fever, (**B**) SIRS and (**C**) sepsis. UW, urine white blood cell; UNIT, urine nitrite; UC, urine culture; UpH, urine pH; Ca, serum calcium.

**Fig.2:**
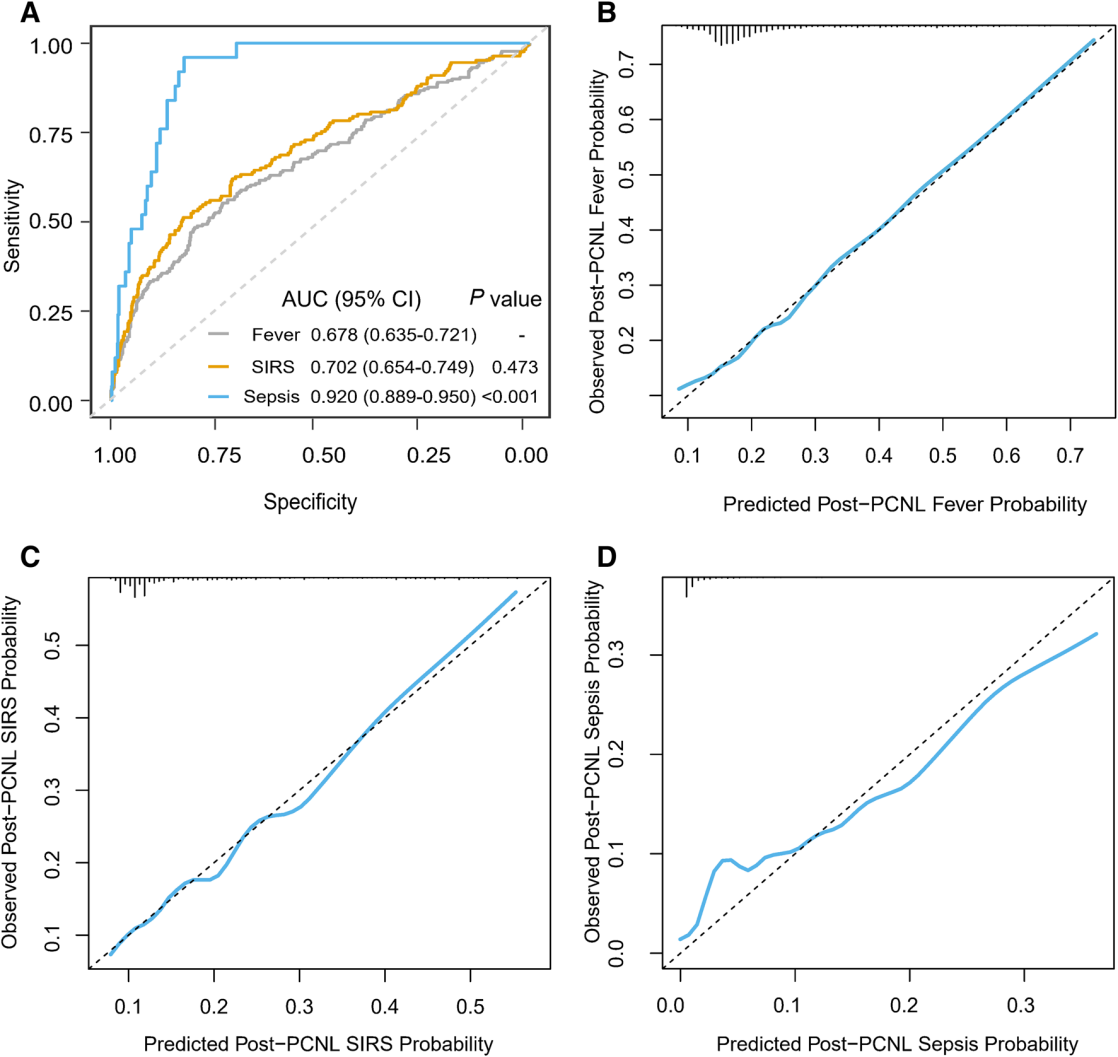
ROC plot for post-PCNL fever, SIRS and sepsis. The C-index of sepsis is significantly higher than SIRS (*P* < 0.001), but there is no significance between SIRS C-index and fever (*P* = 0.473) (**A**); Calibration plots for post-PCNL (**B**) fever, (**C**) SIRS and (**D**) sepsis.

**Fig.3:**
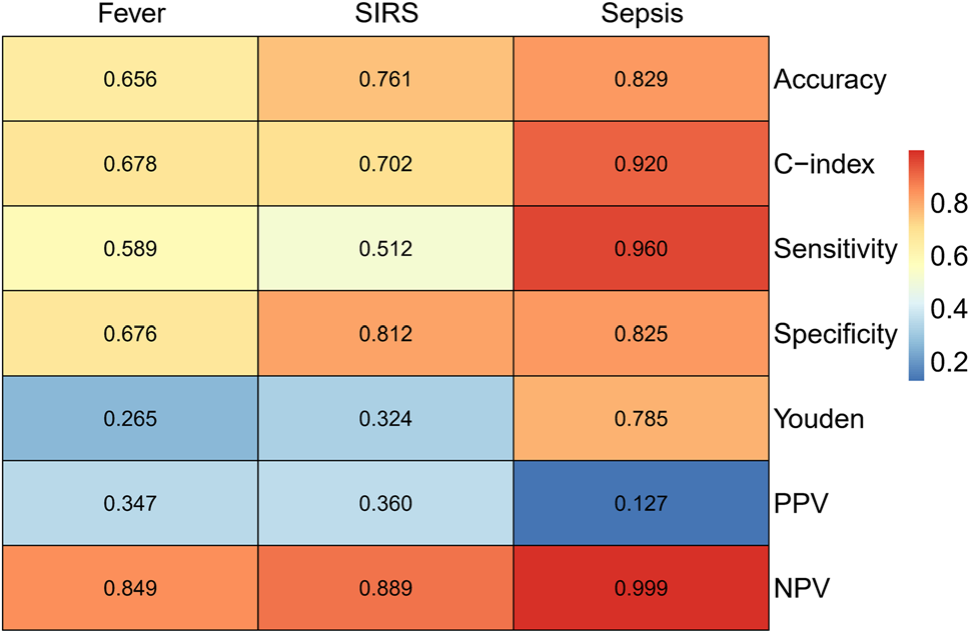
Heat map displays evaluation of the post-PCNL fever, SIRS and sepsis. C-index, concordance index; PPV, positive predictive value; NPV, negative predictive value.

We selected the significant variables in the multivariate logistic regression to establish nomograms showed in Fig. 1. Each variable corresponding to these nomograms was scored points based on its contribution to the model. Total points for each nomogram corresponded to the risk and predicted the likelihood of an outcome. Figure 2A showed their ROCs respectively. The C-index of fever was 0.678 (95% CI 0.635–0.721) and the C-index of SIRS was 0.702 (95%CI 0.654–0.749). Interestingly, the C-index of the sepsis was 0.920 (95% CI 0.889–0.950) which was significantly higher than those of the previous two models. The visualization of the calibration curve as goodness-of-fit test showed that the predicted values of these three models were in agreement with the observed values in Fig. 2B–D. We summarized the evaluation of the three models into a heat map in Fig. 3. Notably, as the severity of these three diseases increased, the most evaluation parameters were improving. Among them, a few parameters were not increased, and it was related to the lower number of sepsis cases in the whole.

**Fig.4:**
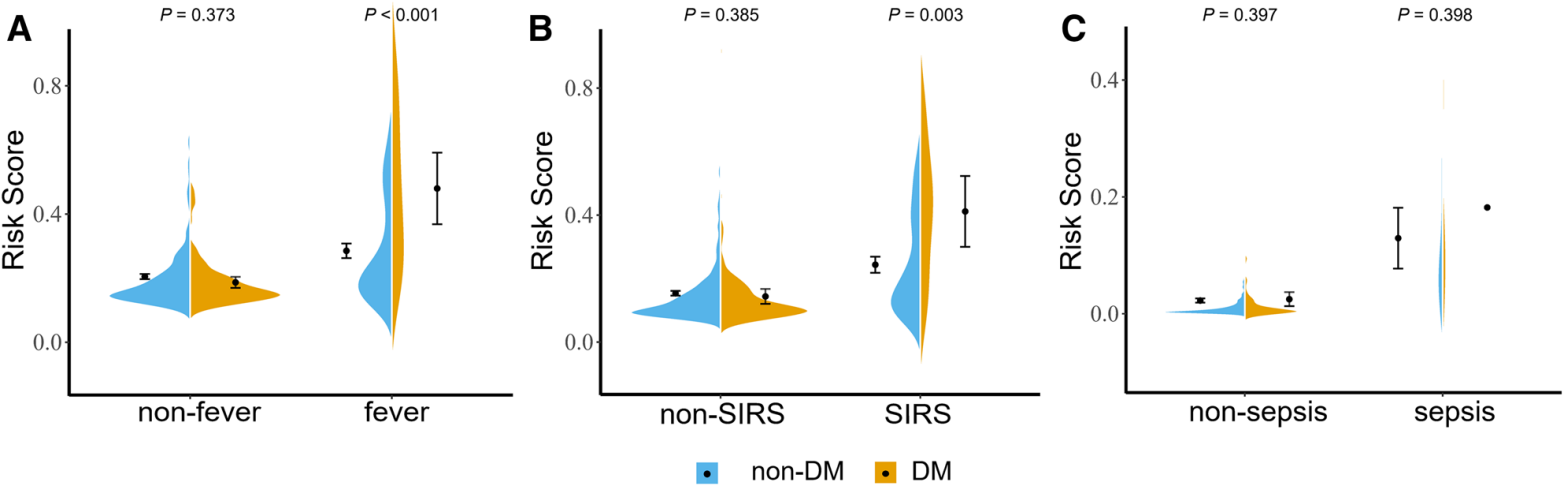
Violinplot of the risk score in the post-PCNL (**A**) fever, (**B**) SIRS and (**C**) sepsis were shown in different DM attribution. The univariate analysis for risk score was applied by using the Mann–Whitney U test. *DM* diabetes mellitus.

With the emergence of severe complications such as sepsis, the predictive ability of the model became stronger, even though the number of positive cases of sepsis was far less than that of fever and SIRS. (Fig. 3: The prediction accuracy of the sepsis model was 0.829, which was higher than that of the other two models; Fig. 2: The C-index of the sepsis model was 0.920, which was higher than that of the other two models). This result suggested that sepsis was more accurate and meaningful than fever and SIRS in predicting the risk of post-PCNL. Additionally, we developed an online dynamic nomogram application, which can dynamically predict post-PCNL sepsis rates (https://www.shinyapps.io/) showed in Supplement Fig. S2. Finally, we found that DM was not directly related to the infection and it influenced outcome indirectly as effect modifier. So, we compared the risk scores of the three groups by DM distribution displayed in Fig. 4. We found that the average risk score of DM was higher than that of non-DM in the positive case groups of the three models, whereas there was no significance in the negative case groups. Due to the small sample size of the sepsis group, the significance was not very high in the group but it cannot be denied that this mechanism did exist in sepsis as the sample size increases.

## Discussion

Previous studies established models to explore the relationship between preoperative examination and fever or SIRS or sepsis after PCNL. We obtained a large sample of PCNL studies within one year, and stratified infection complications to establish visual models. Then we observed the relationship between the models and explained the similarities and differences between them.

Regardless of the severity of complications, UNIT = 2 was a risk factor in all models, and the ORs were similar between models. We suggested that UNIT played a crucial role in the occurrence of postoperative infection of PCNL. Omar et al. have provided evidence that UNIT can accurately predict urine culture and stone culture (sensitivity: 0.92, specificity: 0.98)^12^. UNIT is transformed from nitrate through the action of some bacteria^20^. It is suggested that there are Gram-negative bacteria in urine, especially Escherichia coli. UNIT = 2 indicated a higher concentration of nitrite in urine, because the Gram-negative bacteria with stronger virulence were more active and the infection in patients were more serious. The major difference between the sepsis model and the other two models was UpH and serum Ca. We believed that UpH and Ca could help predict the severity of infection when UNIT = 2 identified the source of serious infection in the body. According to the normal acidification ability of the kidney, urine pH increased with the occurrence of infection and renal tubular acidosis. When infection occurs, Gram-negative bacilli use urease to act on urea to produce alkaline substances such as ammonia and CO_2_ products. At the same time, the alkaline environment is conducive to the reproduction, invasion and release of endotoxin of gram-negative bacilli^21^. When renal tubular acidosis onsets, the kidney has difficulties in the secretion of hydrogen ions in urine and the reabsorption of bicarbonate ions, which eventually leads to the alkalization of urine and the reduction of the secretion of citrate and metabolic acidosis^21^. Reduction of serum calcium is a manifestation of the disorder of calcium channels in cells. Animal experiments found calcium channel disorder in the organs of septic mice with extracellular calcium influx and the significant increase of calcium concentration. As an important signal medium, increased intracellular concentration of calcium ions leads to decreased contraction of cardiovascular smooth muscle cells, disorder of glycogen decomposition and gluconeogenesis of muscle cells and liver cells, and decreased immune response ability of T lymphocytes^22^. The progress of sepsis associated with these two markers is seen as complex chain reaction including metabolic and immune disorder, which causes a further perpetuated homeostatic perturbation^23^.

The study provides new insights that the predictability of the models is improved as the severity of infection increases after PCNL (Fig. 3). Bozkurt et al. believed that leukocytosis and fever were two non-specific indicators of infection, which might be part of the normal physiological response of surgery, and had a poor correlation with preoperative parameters^24^. Although the terms of SIRS and sepsis are related to white blood cells, it does not mean that SIRS and sepsis are common. From leukocytosis to fever, SIRS or sepsis, the threshold is getting higher and higher. The reason why this situation exists depends on the homeostasis of the patient. In general, even if the patient develops leukocytosis or fever, the homeostasis remains good and it is difficult to deteriorate. In severe cases, due to poor homeostasis maintenance, the patients eventually developed into sepsis. This homeostasis is essentially a good performance of preoperative parameters.

Moreover, we also found that SIRS and sepsis were related to the operation time. This may be due to the long operation time leading to long-term infusion of infectious substances released during the operation^25^. In current study, it may be that the body and the infused liquid have been in heat exchange due to the long operation, so the body temperature is seriously lost. Therefore, as long as the risk of infection in the body is low, prolonged surgery will not increase the risk of high fever.

At present, there is still controversy about the association between diabetes and post-PCNL infection. Some studies believed that there was a connection between them, but some researchers noted that the connection was not obvious^26,27^. Interestingly, from the data, we synthesized their views and found that diabetes did not directly affect the incidence of post-PCNL infection (Supplement Tables S2, S4 and S6), but the results were significant when the risk scores were compared in groups (Fig. 4). We believe that diabetes, as an effect modifier, indirectly affects the pre-operative homeostasis and leads to post-PCNL infection. Briefly, this is mainly due to the reduction of immunity and the proliferation of urinary tract bacteria caused by diabetes^28^.

Sepsis prediction was relatively accurate only with preoperative examinations, whereas fever could not be accurately predicted with preoperative examination compared to sepsis. We supposed that post-PCNL fever was caused not only by preoperative factors but also intraoperative and postoperative ones. The occurrence of sepsis mostly related to the patients' homeostasis, meanwhile the risk factors of complications such as fever and SIRS were relatively superficial. Accordingly, we suggest to establish the nomogram to predict Post-PCNL sepsis. Nevertheless, its inconvenience may still restrict its clinical application. Therefore, we developed an online dynamic nomogram application based on the nomogram of Post-PCNL sepsis. The application can achieve good visualization and dynamically predict the incidence of sepsis in postoperative patients. Finally, as a supplement for the nomogram, comparison in groups was generated to distinguish patients at different risks of DM attribution.

Our study has several limitations. First, our research was conducted in a single center. Although the whole sample size was not small, the number of positive cases was still not enough, especially sepsis. Further external validation from another data is needed. Second, we did not include non-fever as a predictor because false negatives would decrease its predictability as postoperative routine urine test was not detected immediately. Third, this retrospective study has inevitable deviation. Some of the preoperative variables have been missed and not included in the study. In the next step, we will improve the quality of the data and cooperate with multiple hospitals to generate more accurate models.

## Conclusion

To summarize, the comparison between the models shows that the prediction of sepsis based on preoperative examination is more accurate than that of fever and SIRS. The incidence of sepsis is a process of preoperative homeostatic perturbation. This is the first development of an online dynamic nomogram application that can optimize individual treatment and follow-up strategies for patients with high risks of sepsis.

## Supplementary information

Supplementary file1

## Data Availability

The datasets generated during and/or analysed during the current study are available from the corresponding author on reasonable request. Additional information and data is available from the corresponding author on reasonable request.
