# Supplementary material for: Predicting and comparing postoperative infections in different stratification following PCNL based on nomograms
Source: Sci Rep. 2020 Jul 9;10:11337. doi: 10.1038/s41598-020-68430-3 (PMC7347600; doi:10.1038/s41598-020-68430-3)
Supplement: Supplementary file 1 — Supplementary file1 [file 41598_2020_68430_MOESM1_ESM.pdf]

Supplementary Information

Predicting and comparing postoperative infections in different stratification following PCNL based on nomograms

Enyan Jiang§, Haixiang Guo§, Bowei Yang§, Pei Li§, Prashant Mishra§, Tongxin Yang§, Yuhang Li§, Haifeng Wang§, Yongming Jiang§ *

§ The Second Affiliated Hospital of Kunming Medical University, Yunnan Urology Speciality Hospital, Yunnan Key Laboratory of Urology, Kunming, Yunnan, P. R. China

***Corresponding author:**
Yongming Jiang Ph.D.
E-mail: drjyml@163.com
ORCID iD: https://orcid.org/0000-0002-8281-6032
Tel: 86 13888503805

The Second Affiliated Hospital of Kunming Medical University, Yunnan Urology Speciality Hospital, Yunnan Key Laboratory of Urology, Kunming, Yunnan, P. R. China

Keywords:
Percutaneous nephrolithotomy, risk prediction, models comparison, infection complications, nomograms

In order to research the differences between the mechanisms of infection, we established the three post-PCNL infection models. We used preoperative data as comprehensively as possible to establish models. Some details are not shown specifically in manuscript but described in this supplementary information.

Not including a large number of intraoperative variables

Our research incorporated mainly preoperative variables, which was related to our research purpose. Not including a large number of intraoperative variables did not mean that these were not important, and also lead our evaluation of nomograms don't look perfect. However, we can find some important points from the differences between the models made up of preoperative variables. Comparing these models, we suggested that the more severe the infection was, the more accurate the model predicted. This may be related to the special mechanism of infection, namely preoperative homeostasis.

Establishing multivariate logistic regression

From Dec 5, 2016 to Dec 25, 2017, demographic data and clinical characteristics of 969 cases were displayed in Table S1. The variables in the univariate model whose significance was less than 0.05 could be included in the multivariate logistic regression. The selection of the prediction model was performed with a backward step down selection process with Akaike information criterion (AIC), depicted in Table S2, S4and S6 Variance Inflation Factor (VIF) as a parameter to measure multiple linear regression models displayed in Table S3, S5and S7. Finally, variables with p-values less than 0.05 in multivariate regression were included in the nomogram.

We summarized the evaluation of the three models into a heat map in Fig. S1. Notably, as the severity of these three diseases increased, the most evaluation parameters were improving. Among them, a few parameters were not increased, and it was related to the lower number of sepsis cases in the whole.

Developing an online dynamic nomogram application to predict the post-PCNL sepsis

As a visualization tool, nomograms can help clinicians understand and predict the occurrence of diseases more intuitively, it is still inconvenient to manually calculate each patient. Online dynamic nomograms application only require clinicians to simply enter the relevant parameters of the patient to get the risk of the disease, and this app can be used on the

mobile phone anytime, anywhere.

From the concept of our study, it made more sense to predict post-PCNL sepsis and then we established an online dynamic nomogram application to predict the post-PCNL sepsis patients (https://celeste-chen.shinyapps.io/DynNomapp/) (Fig. S2). The calculator predicted the probability of patients according to their clinical characteristics. For example, the post-PCNL sepsis probability was approximately 65.0% (95% CI 36.4–85.7%) for the patient with UNIT = 2, UC = 1, UpH = 7, Ca = 2, operation time = 180 min evaluated using the online dynamic nomogram.

Discussion on DM (diabetes mellitus) in groups

Our study used comorbidity as a variable for univariate logistic regression. In univariate logistic regression, it was found that DM had no effect on the three models. It excluded the possibility that DM was an independent risk factor, but from the clinical experience, the occurrence of infection was often strongly related to DM, so we conducted an in-depth analysis for DM and found that it, as a special variable, increased the incidence of infection by affecting preoperative variables.

DM may be a confounder or effect modifier. But DM had no significant effect in univariate logistic regression, we excluded the possibility of being confounder, which was considered as an effect modifier to increase the incidence of infection by affecting preoperative variables indirectly. Limited by the number of positive samples, we only had a discussion in groups. We grouped the probability obtained by *predict (model)* as a risk score for Mann-Whitney U test analysis in Fig. S3, and found that the average risk score of DM was higher than that of non-DM in the positive case groups of the three models, while there was no significant in the negative case groups. DM may statistically influenced the preoperative variables as an effect modifier. In practice, DM did influence the urine environment and human immunity, which lead to increase the proliferation of urine bacteria and the risk of infection. Although this difference was not very significant in the third group, but it can’t be denied that this mechanism did exist in sepsis. On the contrary, we speculated that the mechanism also existed in sepsis and positive results appeared as the sample size increased. We also illustrated this problem in the dissection and limit of the manuscript.

Choice between FUTI and non-FUTI

In the design stage, we included post-PCNL variables: moderate SIRS and heavy sepsis. When considering mild complications, we hesitated to choose FUTI or non-FUTI. According

to the Kaygisiz et al. reporting, we found that non-FUTI was a milder complication of infection, which was more common than FUTI. After counting, we found that there were three types of post-PCNL patients: 1.normal, 2.non-FUTI, 3.FUTI. Similar to the Kaygisiz reported, our C-index was 0.791(95%CI 0.758-0.824), when comparing between 2 and 3. But in reality, there are normal people, not just these two types of people. Moreover, 2 was very affected by the postoperative detection interval. If detected immediately after surgery, the proportion of 2 will be high. Furthermore, our purpose is not to distinguish between 2 and 3, so we only need to choose one of mild infection complication 2 or 3. Our results were not very satisfactory, which may be related to our postoperative routine urine testing was performed in the morning after the operation, not immediately. Because our test interval may lead to false negatives and comparing between FUTI and non-FUTI was not our purpose of this report, weighing the pros and cons, we included 3 as a mild postoperative infection complication.

Table S1 Demographic and clinic data of post-PCNL fever, SIRS and sepsis

Characteristic	level	Non-fever	Fever	*P*	Non-SIRS	SIRS	*P*	Non-sepsis	Sepsis	*P*
n		750	219		803	166		944	25	
Gender, n(%)	Female	206 (27.5)	101 (46.1)	<0.001*	233 (29.0)	74 (44.6)	<0.001*	290 (30.7)	17 (68.0)	<0.001*
	Male	544 (72.5)	118 (53.9)		570 (71.0)	92 (55.4)		654 (69.3)	8 (32.0)	
Age, y(median [IQR])		50.00 [41.00, 58.00]	50.00 [42.50, 56.00]	0.996	50.00 [41.00, 58.00]	50.00 [41.00, 57.00]	0.786	50.00 [41.00, 58.00]	52.00 [45.00, 58.00]	0.361
BMI, kg/m^2(median [IQR])		23.51 [21.09, 25.76]	23.44 [21.18, 25.65]	0.976	23.49 [21.10, 25.71]	23.58 [20.91, 25.90]	0.704	23.51 [21.10, 25.71]	22.22 [20.00, 25.91]	0.559
Hypertension, n(%)	No	583 (77.7)	169 (77.2)	0.933	625 (77.8)	127 (76.5)	0.786	731 (77.4)	21 (84.0)	0.593
	Yes	167 (22.3)	50 (22.8)		178 (22.2)	39 (23.5)		213 (22.6)	4 (16.0)	
DM, n(%)	No	669 (89.2)	197 (90.0)	0.846	717 (89.3)	149 (89.8)	0.968	844 (89.4)	22 (88.0)	0.742†
	Yes	81 (10.8)	22 (10.0)		86 (10.7)	17 (10.2)		100 (10.6)	3 (12.0)	
UR, cells/μL(median [IQR])		64.25 [20.83, 308.07]	78.10 [21.50, 300.80]	0.484	62.00 [20.45, 308.55]	102.25 [24.65, 292.15]	0.148	65.25 [20.88, 304.85]	149.50 [57.30, 507.70]	0.037*
UW, cells/μL(median [IQR])		0.12 [0.05, 0.44]	0.42 [0.08, 2.22]	<0.001*	0.12 [0.05, 0.46]	0.59 [0.12, 2.41]	<0.001*	0.14 [0.05, 0.58]	1.68 [0.96, 2.95]	<0.001*
UB, cells/μL(median [IQR])		59.90 [27.00, 296.80]	248.00 [39.80, 2534.60]	<0.001*	62.30 [27.30, 304.40]	359.00 [40.27, 3637.85]	<0.001*	69.10 [27.90, 481.88]	2304.50 [661.80, 7287.10]	<0.001*
UNIT, n(%)	0	673 (89.7)	149 (68.0)	<0.001*†	715 (89.0)	107 (64.5)	<0.001*†	811 (85.9)	11 (44.0)	<0.001*†
	1	16 (2.1)	6 (2.7)		17 (2.1)	5 (3.0)		21 (2.2)	1 (4.0)	
	2	61 (8.1)	64 (29.2)		71 (8.8)	54 (32.5)		112 (11.9)	13 (52.0)	
UpH (median [IQR])		6.00 [5.50, 6.50]	6.00 [5.50, 6.50]	0.102	6.00 [5.50, 6.50]	6.00 [5.50, 6.50]	0.124	6.00 [5.50, 6.50]	6.50 [6.00, 6.50]	<0.001*
UC, n(%)	Negative	604 (80.5)	129 (58.9)	<0.001*	643 (80.1)	90 (54.2)	<0.001*	727 (77.0)	6 (24.0)	<0.001*

	Positive	146 (19.5)	90 (41.1)		160 (19.9)	76 (45.8)		217 (23.0)	19 (76.0)	
WBC, cells × 10^9/L(median [IQR])		6.71 [5.57, 7.88]	6.51 [5.46, 7.89]	0.502	6.70 [5.54, 7.89]	6.60 [5.59, 7.81]	0.902	6.71 [5.55, 7.89]	6.07 [5.17, 7.53]	0.113
N, cells × 10^9/L(median [IQR])		3.72 [3.02, 4.76]	3.77 [2.90, 4.73]	0.634	3.71 [3.00, 4.78]	3.83 [3.04, 4.68]	0.779	3.72 [3.01, 4.77]	3.78 [2.92, 4.27]	0.536
L, cells × 10^9/L(median [IQR])		2.12 [1.70, 2.57]	2.06 [1.58, 2.51]	0.072	2.11 [1.69, 2.57]	2.06 [1.58, 2.50]	0.107	2.09 [1.67, 2.56]	1.98 [1.55, 2.45]	0.29
PLT, cells × 10^9/L(median [IQR])		234.00 [194.25, 279.75]	249.00 [207.00, 303.50]	0.009*	234.00 [195.00, 281.50]	253.00 [208.50, 306.50]	0.008*	236.00 [196.00, 287.00]	245.00 [222.00, 323.00]	0.296
HGB, cells × 10^12/L(median [IQR])		148.00 [135.00, 161.00]	139.00 [126.00, 154.00]	<0.001*	147.00 [134.00, 161.00]	140.00 [126.00, 152.00]	<0.001*	146.00 [133.00, 160.00]	127.00 [115.00, 142.00]	<0.001*
ALB, g/L(median [IQR])		44.20 [41.30, 46.60]	43.10 [41.00, 46.10]	0.040*	44.10 [41.40, 46.60]	43.25 [40.52, 46.10]	0.042*	44.10 [41.30, 46.50]	42.20 [37.60, 44.70]	0.005*
Cr, μmol/L(median [IQR])		84.50 [72.00, 100.75]	80.00 [69.00, 102.00]	0.139	84.00 [72.00, 101.00]	80.00 [69.25, 102.75]	0.233	84.00 [72.00, 100.00]	93.00 [77.00, 133.00]	0.119
UA, μmol/L(median [IQR])		399.50 [338.50, 469.00]	385.00 [325.50, 458.00]	0.074	398.00 [336.50, 466.50]	390.00 [327.00, 468.00]	0.504	399.00 [336.00, 468.00]	351.00 [301.00, 412.00]	0.039*
GLU, mmol/L(median [IQR])		5.26 [4.85, 5.81]	5.23 [4.81, 5.83]	0.667	5.25 [4.85, 5.83]	5.25 [4.83, 5.77]	0.718	5.25 [4.84, 5.81]	5.51 [5.03, 6.03]	0.31
K, mmol/L(median [IQR])		4.05 [3.83, 4.26]	4.01 [3.76, 4.25]	0.199	4.05 [3.83, 4.26]	4.00 [3.75, 4.25]	0.141	4.04 [3.83, 4.26]	3.97 [3.57, 4.22]	0.087

Ca, mmol/L(median [IQR])		2.30 [2.23, 2.38]	2.30 [2.23, 2.38]	0.776	2.30 [2.23, 2.38]	2.29 [2.22, 2.39]	0.613	2.30 [2.23, 2.38]	2.28 [2.11, 2.34]	0.007*
P, mmol/L(median [IQR])		1.12 [1.00, 1.24]	1.16 [1.02, 1.30]	0.005*	1.13 [1.00, 1.24]	1.16 [1.02, 1.30]	0.037*	1.13 [1.01, 1.26]	1.09 [0.99, 1.23]	0.519
Mg, mmol/L(median [IQR])		0.86 [0.81, 0.92]	0.87 [0.83, 0.92]	0.181	0.87 [0.82, 0.92]	0.87 [0.83, 0.92]	0.46	0.87 [0.82, 0.92]	0.86 [0.81, 0.93]	0.935
Left or Right, n(%)	Left	219 (29.2)	58 (26.5)	0.631	230 (28.6)	47 (28.3)	0.973	269 (28.5)	8 (32.0)	0.45
	Right	204 (27.2)	58 (26.5)		218 (27.1)	44 (26.5)		258 (27.3)	4 (16.0)	
	Bilateral	327 (43.6)	103 (47.0)		355 (44.2)	75 (45.2)		417 (44.2)	13 (52.0)	
Position, n(%)	K	451 (60.1)	152 (69.4)	0.046*†	484 (60.3)	119 (71.7)	0.044*†	583 (61.8)	20 (80.0)	0.395†
	U	51 (6.8)	12 (5.5)		56 (7.0)	7 (4.2)		63 (6.7)	0 (0.0)	
	K-U	240 (32.0)	52 (23.7)		254 (31.6)	38 (22.9)		287 (30.4)	5 (20.0)	
	K-B	4 (0.5)	3 (1.4)		5 (0.6)	2 (1.2)		7 (0.7)	0 (0.0)	
	K-U-B	4 (0.5)	0 (0.0)		4 (0.5)	0 (0.0)		4 (0.4)	0 (0.0)	
Size, cm(median [IQR])		1.30 [0.80, 1.90]	1.20 [0.90, 1.90]	0.665	1.20 [0.80, 1.90]	1.25 [0.90, 1.90]	0.586	1.20 [0.80, 1.83]	1.50 [1.10, 2.50]	0.046*
CT-Hu, H(median [IQR])		895.00 [674.25, 1105.75]	907.00 [655.00, 1079.00]	0.464	897.00 [677.50, 1105.00]	891.00 [648.75, 1083.00]	0.317	894.00 [671.75, 1104.25]	976.00 [681.00, 1061.00]	0.866
Staghorn, n(%)	No	613 (81.7)	164 (74.9)	0.032*	656 (81.7)	121 (72.9)	0.013*	762 (80.7)	15 (60.0)	0.019*†
	Yes	137 (18.3)	55 (25.1)		147 (18.3)	45 (27.1)		182 (19.3)	10 (40.0)	
Hydronephrosis, n(%)	No	54 (7.2)	9 (4.1)	0.442	59 (7.3)	4 (2.4)	0.093	61 (6.5)	2 (8.0)	0.839†
	Mild	488 (65.1)	146 (66.7)		516 (64.3)	118 (71.1)		619 (65.6)	15 (60.0)	
	Moderate	169 (22.5)	52 (23.7)		186 (23.2)	35 (21.1)		214 (22.7)	7 (28.0)	
	Severe	39 (5.2)	12 (5.5)		42 (5.2)	9 (5.4)		50 (5.3)	1 (4.0)	

Operation time, min (median [IQR])		100.00 [75.00 , 125.00]	110.00 [84.50 , 125.00]	0.057	100.00 [75.00 , 125.00]	115.00 [90.00 , 138.75]	0.003*	100.00 [75.00 , 125.00]	120.00 [95.00 , 170.00]	0.029*
Sheath, n(%)	18Fr	16 (2.1)	6 (2.7)	0.503†	16 (2.0)	6 (3.6)	0.515†	22 (2.3)	0 (0.0)	0.748†
	20Fr	167 (22.3)	43 (19.6)		178 (22.2)	32 (19.3)		206 (21.8)	4 (16.0)	
	22Fr	436 (58.1)	138 (63.0)		474 (59.0)	100 (60.2)		559 (59.2)	15 (60.0)	
	24Fr	131 (17.5)	32 (14.6)		135 (16.8)	28 (16.9)		157 (16.6)	6 (24.0)	
Multiple calculi, n(%)	No	640 (85.3)	191 (87.2)	0.555	687 (85.6)	144 (86.7)	0.781	811 (85.9)	20 (80.0)	0.385†
	Yes	110 (14.7)	28 (12.8)		116 (14.4)	22 (13.3)		133 (14.1)	5 (20.0)	

Mann-Whitney U test for comparison between non-normal data, chi-square test for comparison between categorical data

† Values indicate using Fisher's exact probability method

* Values indicate statistically significant (P < 0.05)

Abbreviations: BMI, body mass index; DM, diabetes mellitus; UR, urine red blood cell; UW, urine white blood cell; UB,urine bacteria; UNIT, urine nitrite; UpH, urine pH; UC, urine culture; HGB, hemoglobin; WBC, serum white blood cell; N, serum neutrophils; L, serum lymphocytes; PLT, platelet; ALB, albumin; Cr, creatinine; UA, uric acid; GLU, fast blood sugar; K, serum potassium; Ca, serum calcium; P, serum phosphorus; Mg, serum magnesium; K-U-B, kidney-ureter-bladder; CT-HU, computed tomography hounsfield unit; SIRS, systemic inflammatory response syndrome.

Table S2 Risk Factors of Post-PCNL Fever

Characteristic		Univariate Logistic Regression Crude OR(95% CI)	*P*	Mulvariate Logistic Regression Adjusted OR(95% CI)	*P*
Gender, male		0.442(0.324,0.604)	< 0.001*		
Age, year		0.999(0.987,1.011)	0.872		
BMI, kg/m^2		1.002(0.960,1.046)	0.924		
Hypertension, yes		1.033(0.716,1.471)	0.860		
DM, yes		0.922(0.549,1.491)	0.750		
UR, cells/µL		1(1,1)	0.602		
UW, cells × 10^3 /µL		1.189(1.118,1.272)	< 0.001*	1.087 (1.021,1.156)	0.006*
UB, cells/µL		1(1,1)	< 0.001*		
UNIIT	0	Ref.	< 0.001*	Ref.	< 0.001*
	1	1.694(0.652,4.401)	0.279†	0.987 (0.358,2.718)	0.979†
	2	4.739(3.199,7.021)	< 0.001*†	2.546 (1.582,4.097)	< 0.001*†
UpH		1.229(0.952,1.583)	0.112		
UC, yes		2.886(2.085,3.991)	< 0.001*	1.480 (0.994,2.204)	
WBC, cells × 10^9/L		0.977(0.901,1.056)	0.562		
N, cells × 10^9/L		0.994(0.904,1.087)	0.893		
L, cells × 10^9/L		0.809(0.646,1.007)	0.062		
PLT, cells × 10^9/L		1.003(1.001,1.005)	0.002*		
HGB cells × 10^{12}/L		0.980(0.973,0.988)	< 0.001*	0.992 (0.983,1.002)	0.055
ALB, g/L		1.006(0.992,1.024)	0.367		
Cr, µmol/L		0.999(0.996,1.002)	0.557		
UA, µmol/L		0.999(0.997,1)	0.105		
GLU, mmol/L		0.952(0.836,1.070)	0.433		
K, mmol/L		0.873(0.583,1.296)	0.504		
Ca, mmol/L		0.878(0.257,2.955)	0.835		
P, mmol/L		2.815(1.349,5.892)	0.006*	2.045 (0.934,4.478)	0.075
Mg, mmol/L		2.092(0.34,13.134)	0.428		
Size, cm		0.992(0.848,1.148)	0.915		
CT-Hu, H		1(0.999,1)	0.529		
Staghorn, yes		1.501(1.044,2.136)	0.026*	1.457 (0.998,2.127)	0.055
Hydronephrosis	None	Ref.	0.398		
	Mild	1.795(0.866,3.723)	0.116†		
	Moderate	1.846(0.854,3.992)	0.119†		
	Severe	1.846(0.709,4.809)	0.209†		
Operation time, min		1.003(1,1.006)	0.076		
Sheath size	18Fr	Ref.	0.505		
	20Fr	0.687(0.254,1.860)	0.460†		
	22Fr	0.844(0.324,2.199)	0.729†		
	24Fr	0.651(0.236,1.797)	0.408†		
Position	Kidney	Ref.	0.036*		
	Ureter	0.698(0.363,1.344)	0.282†		
	Kidney-Ureter	0.643(0.452,0.914)	0.014*†		
	Kidney-Ureter	2.225(0.492,10.055)	0.299†		
	Kidney-Ureter-Bladder	0(0, Inf.)	0.976†		
Left or Right	Left		0.631		
	Right	1.074(0.712,1.619)	0.735†		
	Bilateral	1.189(0.826,1.712)	0.351†		
Multiple calculi, yes		0.853(0.546,1.331)	0.479		

* Values indicate statistically significant (P < 0.05)

† Values indicate *P* in Wald's-test, others are in LR-test

The appearance of *Inf.* is caused by insufficient sample size

Abbreviations: BMI, body mass index; DM, diabetes mellitus; UR, urine red blood cell; UW, urine white blood cell; UB, urine bacteria; UNIT, urine nitrite; UpH, urine pH; UC, urine culture; HGB hemoglobin; WBC, serum white blood cell; N, serum neutrophils; L, serum lymphocytes; PLT, platelet; ALB, albumin; Cr, creatinine; UA, uric acid; GLU, fast blood sugar; K, serum potassium; Ca, serum calcium; P, serum phosphorus; Mg, serum magnesium, CT-HU, computed tomography hounsfield unit; OR, odds ratio; CI, confidence interval; Inf, infinity; Ref, reference.

Table S3 Generalized Collinearity Diagnostics of Post-PCNL Fever

Characteristic	VIF
UW	1.092
UNIT	1.095
UC	1.174
HGB	1.078
P	1.017
Staghorn	1.006

From this output result, the VIFs of UW, UNIT, UC, HGB, P and Staghorn are approximately 1 and far less than 10, indicating that there is no serious multicollinearity between the six variables.

Abbreviations: UW, urine white blood cell; UNIT, urine nitrite; UC, urine culture; HGB hemoglobin; P, serum phosphorus.

Table S4 Risk Factors of Post-PCNL SIRS

Characteristic		Univariate Logistic Regression		Mulvariate Logistic Regression	
		Crude OR(95% CI)	*P*	Adjusted OR(95% CI)	*P*
Gender, male		0.508(0.361,0.716)	< 0.001*		
Age, year		0.999(0.986,1.012)	0.874		
BMI, kg/m^2		1.016(0.968,1.066)	0.517		
Hypertension, yes		1.078(0.719,1.589)	0.709		
DM, yes		0.951(0.533,1.609)	0.858		
UR, cells/μL		1(1,1)	0.466		
UW, cells × 10^3 /μL		1.155(1.091,1.229)	< 0.001*	1.056 (0.995,1.121)	0.064
UB, cells/μL		1(1,1)	< 0.001*		
UNIIT	0	Ref.	< 0.001*	Ref.	< 0.001*
	1	1.596(0.71,5.437)	0.193 †	1.100 (0.373,3.241)	0.863†
	2	5.082(3.379,7.643)	< 0.001*†	2.880(1.760,4.712)	< 0.001*†
UpH		1.245(0.939,1.646)	0.125		
UC, yes		3.394(2.387,4.821)	< 0.001*	1.887 (1.231,2.893)	0.004*
WBC, cells × 10^9/L		1.004(0.92,1.093)	0.924		
N, cells × 10^9/L		1.024(0.924,1.127)	0.644		
L, cells × 10^9/L		0.814(0.633,1.038)	0.103		
PLT, cells × 10^9/L		1.003(1.001,1.006)	0.002 *	1.002 (0.999,1.004)	0.088
HGB, cells × 10^{12}/L		0.981(0.973,0.989)	< 0.001*		
ALB, g/L		1.007(0.992,1.026)	0.289		
Cr, µmol/L		1(0.996,1.003)	0.952		
UA, µmol/L		1(0.998,1.001)	0.580		
GLU, mmol/L		0.945(0.815,1.077)	0.426		
K, mmol/L		0.745(0.473,1.161)	0.199		
Ca, mmol/L		0.449(0.112,1.751)	0.253		
P, mmol/L		2.743(1.218,6.154)	0.014 *		
Mg, mmol/L		1.309(0.176,9.985)	0.794		
Size, cm		0.988(0.827,1.16)	0.887		
CT-Hu, H		1(0.999,1)	0.352		
Staghorn, yes		1.66(1.12,2.428)	0.010 *	1.484 (0.981,2.246)	0.066
Hydronephrosis	None	Ref.	0.052		
	Mild	3.373(1.202,9.469)	0.021 *†		
	Moderate	2.776(0.947,8.133)	0.063 †		
	Severe	3.161(0.912,10.949)	0.070 †		
Operation time, min		1.005(1.001,1.008)	0.007 *	1.005 (1.001,1.008)	0.012*
Sheath size	18Fr	Ref.	0.576		
	20Fr	0.479(0.174,1.317)	0.154 †		
	22Fr	0.563(0.215,1.473)	0.242 †		
	24Fr	0.553(0.199,1.538)	0.256 †		
Position	Kidney	Ref.	0.035 †		
	Ureter	0.508(0.226,1.144)	0.102		
	Kidney-Ureter	0.608(0.41,0.904)	0.014 *†		
	Kidney-Ureter	1.627(0.312,8.488)	0.564 †		
	Kidney-Ureter-Bladder	0(0, Inf.)	0.976 †		
Left or Right	Left	Ref.	0.973		
	Right	0.988(0.629,1.55)	0.957 †		
	Bilateral	1.034(0.693,1.543)	0.871 †		
Multiple calculi, yes		0.905(0.542,1.451)	0.689		

* Values indicate statistically significant (P < 0.05)

† Values indicate *P* in Wald's-test, others are in LR-test

The appearance of *Inf.* is caused by insufficient sample size

Abbreviations: BMI, body mass index; DM, diabetes mellitus; UR, urine red blood cell; UW, urine white blood cell; UB, urine bacteria; UNIT, urine nitrite; UpH, urine pH; UC, urine culture; HGB, hemoglobin; WBC, serum white blood cell; N, serum neutrophils; L, serum lymphocytes; PLT, platelet; ALB, albumin; Cr, creatinine; UA, uric acid; GLU, fast blood sugar; K, serum potassium; Ca, serum calcium; P, serum phosphorus; Mg, serum magnesium, CT-HU, computed tomography hounsfield unit; OR, odds ratio; CI, confidence interval; Inf, infinity; Ref, reference.

Table S5 Generalized Collinearity Diagnostics of Post-PCNL SIRS

Characteristic	VIF
UW	1.094
UC	1.169
UNIT	1.091
PLT	1.017
Staghorn	1.010
Operation Time	1.007

From this output result, the VIFs of UW, UC, UNIT, PLT, Staghorn and Operation Time are approximately 1 and far less than 10, indicating that there is no serious multicollinearity between the six variables.

Abbreviations: UW, urine white blood cell; UC, urine culture; UNIT, urine nitrite; PLT, platelet.

Table S6 Risk Factors of Post-PCNL Sepsis

		Univariate Logistic Regression		Mulvariate Logistic Regression	
Characteristic		Crude OR(95% CI)	*P*	Adjusted OR(95% CI)	*P*
Gender, male		0.209(0.084,0.475)	< 0.001*	0.446 (0.173,1.149)	0.087
Age, year		1.019(0.987,1.055)	0.263		
BMI, kg/m^2		0.979(0.872,1.097)	0.723		
Hypertension, yes		0.654(0.189,1.74)	0.440		
DM, yes		1.151(0.269,3.397)	0.822		
UR, cells/μL		1(1,1)	0.146		
UW, cells × 10^3 /μL		1.088(1.009,1.164)	0.012*		
UB, cells/μL		1(1,1)	0.006*		
UNIIT	0	Ref.	< 0.001*	Ref.	0.046*
	1	3.511(0.433,28.454)	0.240†	0.816 (0.089,7.488)	0.158†
	2	8.558(3.743,19.565)	< 0.001*†	2.794 (1.06,7.362)	0.038*†
UpH		2.941(1.605,5.32)	< 0.001*	1.985 (1.016,3.879)	0.046*†
UC, yes		10.609(4.423,29.431)	< 0.001*	4.985 (1.713,14.511)	0.002*†
WBC, cells × 10^9/L		0.873(0.683,1.085)	0.252		
N, cells × 10^9/L		0.921(0.686,1.169)	0.548		
L, cells × 10^9/L		0.704(0.372,1.263)	0.261		
PLT, cells × 10^9/L		1.003(0.998,1.007)	0.269		
HGB cells × 10^{12}/L		0.957(0.938,0.976)	< 0.001*		
ALB, g/L		0.891(0.821,0.959)	0.002*		
Cr, μmol/L		1.005(1,1.009)	0.021*		
UA, μmol/L		0.996(0.992,1)	0.076		
GLU, mmol/L		1.061(0.765,1.332)	0.671		
K, mmol/L		0.362(0.125,1.063)	0.065		
Ca, mmol/L		0.003(0,0.073)	< 0.001*	0.004 (0,0.146)	0.003*
P, mmol/L		0.560(0.071,3.949)	0.573		
Mg, mmol/L		0.328(0.003,40.026)	0.643		
Size, cm		1.25(0.893,1.620)	0.132		
CT-Hu, H		1(0.999,1.001)	0.952		
Staghorn, yes		2.791(1.196,6.248)	0.014*	2.417 (0.970,6.021)	0.065
Hydronephrosis	None	Ref.	0.903		
	Mild	0.739(0.165,3.308)	0.693†		
	Moderate	0.998(0.202,4.927)	0.998†		
	Severe	0.610(0.054,6.924)	0.690†		
Operation time, min		1.009(1.002,1.016)	0.008*	1.009(1.001,1.018)	0.034*
Sheath size	18Fr	Ref.	0.519		
	20Fr	826112.854(0,Inf)	0.987†		
	22Fr	1141631.806(0,Inf)	0.987†		
	24Fr	1625916.000(0,Inf)	0.987†		
Position	Kidney	Ref.	0.195		
	Ureter	0(0, Inf.)	0.985†		
	Kidney-Ureter	0.508(0.189,1.367)	0.180†		
	Kidney-Ureter	0(0, Inf.)	0.995†		
	Kidney-Ureter-Bladder	0(0, Inf.)	0.996†		
Left or Right	Left	Ref.	0.413		
	Right	0.521(0.155,1.752)	0.292†		
	Bilateral	1.048(0.429,2.563)	0.918†		
Multiple calculi, yes		1.524(0.501,3.839)	0.407		

* Values indicate statistically significant (P < 0.05)

† Values indicate *P* in Wald's-test, others are in LR-test

The appearance of *Inf.* is caused by insufficient sample size

Abbreviations: BMI, body mass index; DM, diabetes mellitus; UR, urine red blood cell; UW, urine white blood cell; UB, urine bacteria; UNIT, urine nitrite; UpH, urine pH; UC, urine culture; HGB hemoglobin; WBC, serum white blood cell; N, serum neutrophils; L, serum lymphocytes; PLT, platelet; ALB, albumin; Cr, creatinine; UA, uric acid; GLU, fast blood sugar; K, serum potassium; Ca, serum calcium; P, serum phosphorus; Mg, serum magnesium, CT-HU, computed tomography hounsfield unit; OR, odds ratio; CI, confidence interval; Inf, infinity; Ref, reference.

Table S7 Generalized Collinearity Diagnostics of Post-PCNL Sepsis

Characteristic	VIF
Gender	1.050
UNIT	1.054
UpH	1.018
UC	1.110
Ca	1.040
Staghorn	1.021
Operation Time	1.027

From this output result, the VIFs of Gender, UNIT, UpH, UC, Ca, Staghorn, Operation Time and Staghorn are approximately 1 and far less than 10, indicating that there is no serious multicollinearity between the seven variables.

Abbreviations: UNIT, urine nitrite; UpH, urine pH; UC, urine culture; Ca, serum calcium

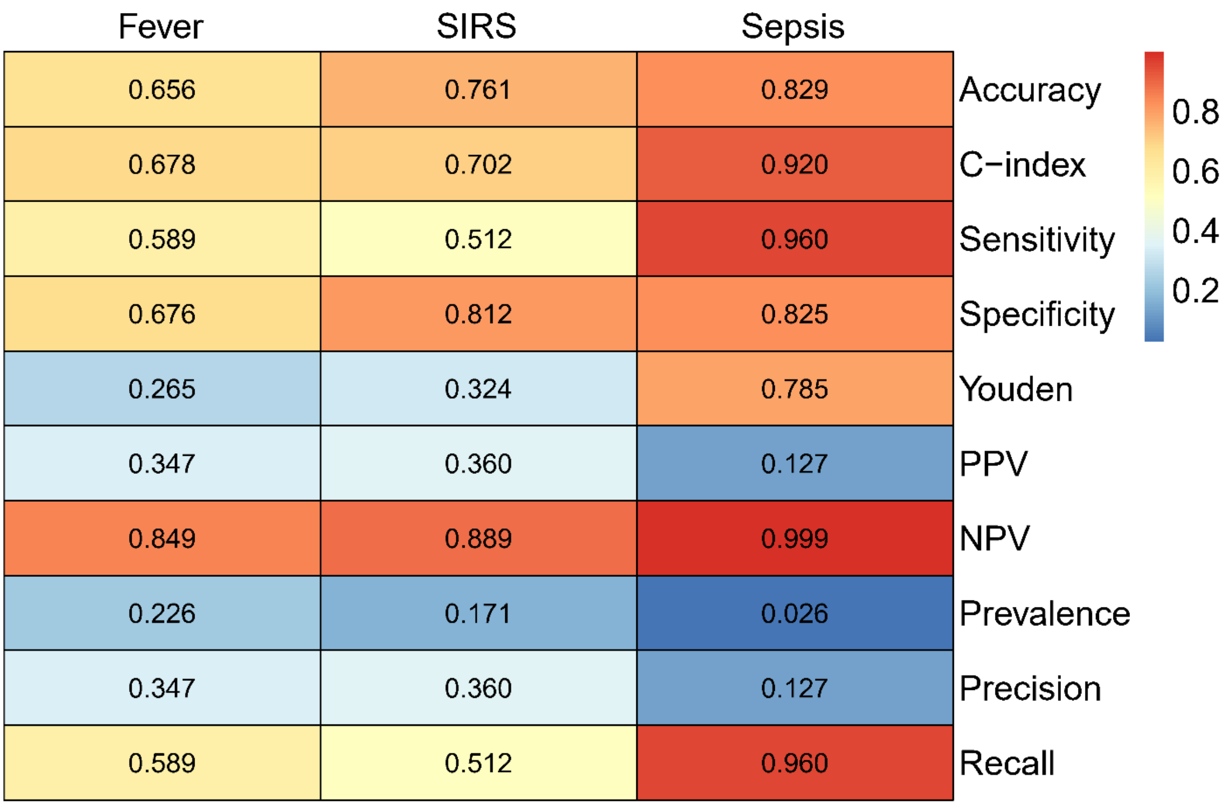

Fig. S1 Heat map displays evaluation of the post-PCNL fever, SIRS and sepsis.

Abbreviations: C-index, concordance index; PPV, positive predictive value; NPV, negative predictive value.

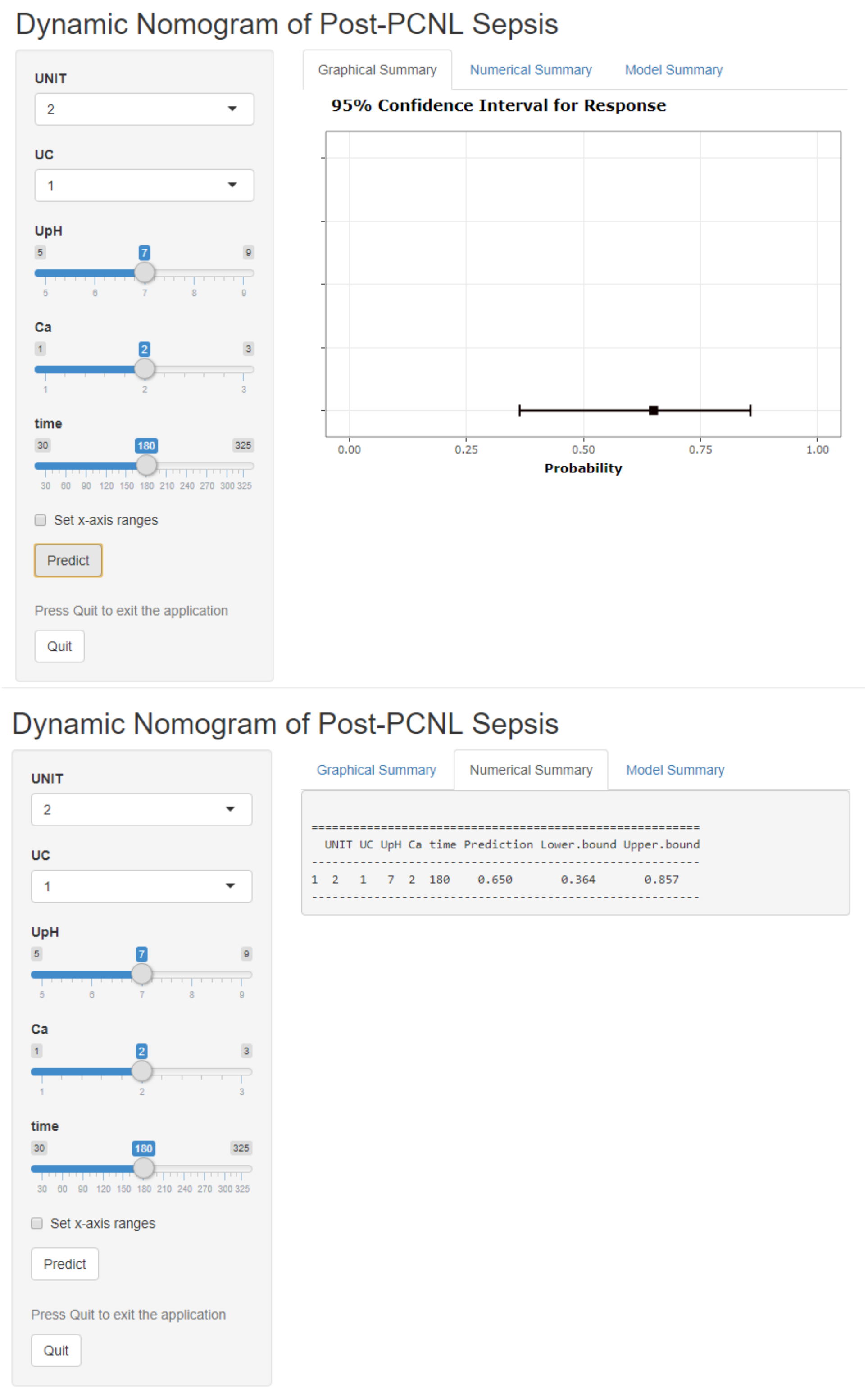

Fig. S2 A patient with UNIT = 2, UC = 1, UpH = 7, Ca = 2, operation time = 180 (min) was evaluated for the risk of post-PCNL sepsis using the online dynamic nomogram.

Abbreviation: UNIT, urine nitrite; UC, urine culture; UpH, urine pH; Ca, serum calcium.

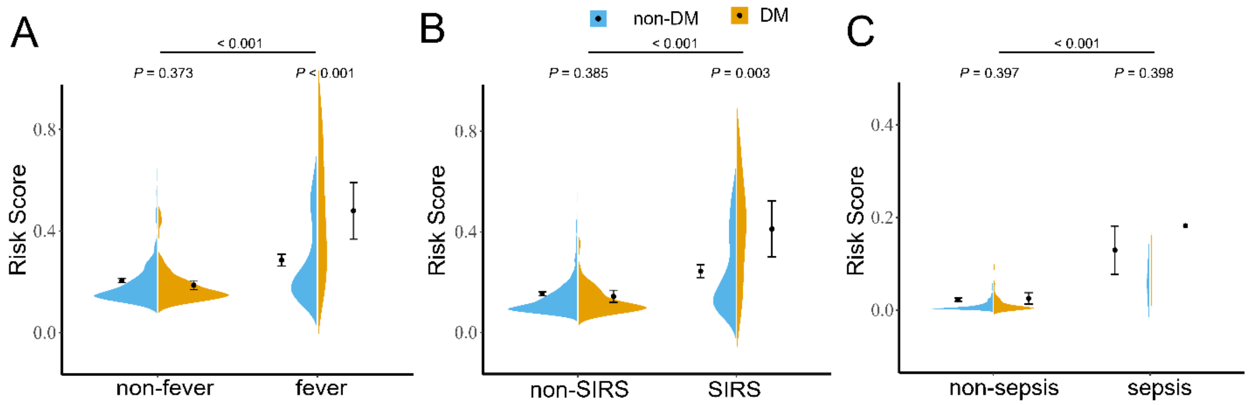

Fig. S3 Violinplots of the risk score in the post-PCNL (A) fever, (B) SIRS and (C) sepsis are shown in different DM attribution.

The univariate analysis for risk score was applied by using the Mann-Whitney U test.

Abbreviations: DM, diabetes mellitus.
